# Crystal structure of the Rab-binding domain of Rab11 family-interacting protein 2

**DOI:** 10.1107/S2053230X20009164

**Published:** 2020-07-28

**Authors:** Aoife Mairead Kearney, Amir Rafiq Khan

**Affiliations:** a School of Biochemistry and Immunology, Trinity College Dublin, 152–160 Pearse Street, Dublin D2, Ireland; b Division of Newborn Medicine, Boston Children’s Hospital, Center for Life Sciences, 3 Blackfan Circle, Boston, MA 02446, USA

**Keywords:** Rab11 family-interacting protein 2, *ab initio* phasing, Rab-binding domain, GTPases

## Abstract

The small GTPases Rab11, Rab14 and Rab25 regulate membrane trafficking through the recruitment of Rab11 family-interacting proteins (FIPs) to endocytic compartments. FIPs are multi-domain effector proteins that have a highly conserved Rab-binding domain (RBD) at their C-termini. Several structures of complexes of Rab11 with RBDs have previously been determined, including those of Rab11–FIP2 and Rab11–FIP3. In addition, the structures of the Rab14–FIP1 and Rab25–FIP2 complexes have been determined. All of the RBD structures contain a central parallel coiled coil in the RBD that binds to the switch 1 and switch 2 regions of the Rab. Here, the crystal structure of the uncomplexed RBD of FIP2 is presented at 2.3 Å resolution. The structure reveals antiparallel α-helices that associate through polar interactions. These include a remarkable stack of arginine residues within a four-helix bundle in the crystal lattice.

## Abstract 2

The structure of the uncomplexed Rab-binding domain of Rab11 family-interacting protein 2 has been determined at 2.3 Å resolution. The structure reveals antiparallel α-helical dimers that associate in the crystal lattice through polar interactions. The dimers assemble into a four-helix bundle in the lattice that is brought together by a remarkable stack of arginine residues.

## 1. Introduction   

Rab GTPases regulate membrane-trafficking pathways in eukaryotic cells *via* the recruitment of effector proteins to subcellular compartments (Hutagalung & Novick, 2011 ▸). Rab11 family-interacting protein 2 (FIP2) is a 512-residue effector that contains a Rab-binding domain (RBD) at its C-terminus. The RBD is shared by a family of effector proteins, which include Rab-coupling protein (RCP or FIP1), FIP2 and FIP3 (Hales *et al.*, 2001 ▸). The N-terminus of this modular effector family is variable and consists of domains that include EF-hands, ERM domains, C2 domains and myosin V-binding domains. These effectors regulate membrane trafficking following their recruitment to subcellular compartments by Rab11, Rab14 and Rab25 GTPases. Interaction with Rabs is facilitated by the RBD, which is highly conserved in sequence and structure. The crystal structures of Rab11–FIP2, Rab11–FIP3 and Rab25–FIP2 complexes revealed that the RBD is a parallel α-helical coiled coil. The dimers of FIP2 and FIP3 are stabilized by hydrophobic interactions, and the symmetric coiled coil binds to two Rab molecules on each side of the dimer.

The effector FIP2 contains an N-terminal C2 domain that binds to phospholipids (residues 15–102; Lindsay & McCaffrey, 2004 ▸), a myosin Vb-binding domain (residues 129–290; Hales *et al.*, 2002 ▸) and a C-terminal RBD (residues 440–512; Hales *et al.*, 2001 ▸) that binds to Rab11. In polarized cells, FIP2 has classically been linked to an endosomal retrieval system that includes cargo such as the transferrin receptor (Lindsay & McCaffrey, 2002 ▸). More recently, FIP2 functions have been associated with critical processes that include synaptic vesicle trafficking (Royo *et al.*, 2019 ▸) and TLR4-mediated phago­cytosis (Skjesol *et al.*, 2019 ▸). Moreover, FIP2 functions have been linked to various cancers (Dong & Wu, 2018 ▸; Dong *et al.*, 2016 ▸; Zhang *et al.*, 2018 ▸).

The adaptor functions of FIP2 linking Rab11 membranes to the cytoskeleton are likely to involve dynamic conformational changes. Here, the crystal structure of an uncomplexed form of FIP2 was determined at 2.3 Å resolution by the *ab initio* phasing method in *ARCIMBOLDO*. In contrast to previous crystal structures of complexes, the structure of isolated FIP2 reveals the formation of antiparallel α-helical dimers that are stabilized by polar interactions.

## 2. Materials and methods   

### 2.1. Macromolecule production   

A 20 ml overnight culture was added to a conical flask containing 1 l sterile 2×YT broth medium along with a 1:1000 dilution of 30 mg ml^−1^ kanamycin. The culture was incubated at 37°C and 180 rev min^−1^ until an OD (*A*_600_) reading between 0.6 and 0.8 was reached. At this point, protein expression was induced with 0.5 m*M* isopropyl β-d-1-thiogalactopyranoside and the temperature of the shaking incubator was adjusted to the optimum temperature for expression of the construct. At 18°C, the culture was left to incubate overnight or for approximately 18 h. At 37°C, the culture was left to incubate for 3 h. To isolate the protein, 20 ml extraction buffer (300 m*M* NaCl, 10 m*M* Tris, 10 m*M* imidazole, 5 m*M* β-mercaptoethanol) was used to resuspend the bacterial pellet from 1 l of culture. The cell pellet was homogenized and the solution was sonicated by a series of 2 min pulses (duty cycle 30%, output 5, Branson sonifier). Each sample was subjected to sonication three times, resting on ice between rounds of sonication. The lysate was spun in a floor centrifuge at 18 000 rev min^−1^ and 4°C for 30 min. The resulting supernatant was applied onto a nickel agarose gravity-flow chromatography column. The column was then washed thoroughly with extraction buffer (300 m*M* NaCl, 10 m*M* Tris, 10 m*M* imidazole, 5 m*M* β-mercaptoethanol). The protein was eluted from the column with elution buffer (300 m*M* NaCl, 10 m*M* Tris, 200 m*M* imidazole, 5 m*M* β-mercaptoethanol). The protein was cleaved with Tobacco etch virus (TEV) protease overnight in a cold room under dialysis with extraction buffer. After cleavage, the protein solution was reapplied onto a nickel agarose gravity-flow chromatography column. The flowthrough from the column, containing the cleaved protein, was collected. The protein was then dialyzed into low-salt buffer (5 m*M* NaCl, 10 m*M* Tris, 5 m*M* β-mercaptoethanol) for 3 h. The protein was applied onto a Mono Q anion-exchange column (GE Life Sciences) and a salt gradient was applied from low salt (5 m*M* NaCl, 10 m*M* Tris, 1 m*M* DTT) to high salt (1 *M* NaCl, 10 m*M* Tris, 1 m*M* DTT). The main resulting peak contained pure, cleaved FIP2, as demonstrated by SDS–PAGE. The purified FIP2 was then run on a Superdex 75 16/60 gel-filtration column (buffer: 150 m*M* NaCl, 10 m*M* Tris, 1 m*M* DTT). The resulting peak was taken and concentrated to >6 mg ml^−1^ for crystallization. The protein concentration was determined from the absorbance at 280 nm using an extinction coefficient of 5960 *M* ^−1^ cm^−1^. Macromolecule-production information is summarized in Table 1 ▸.

### 2.2. Crystallization   

Purified FIP2 was concentrated to approximately 6 mg ml^−1^ prior to crystallization. The lead conditions for crystallization were obtained from a sparse-matrix screen (Structure Screen, Hampton Research) as a combination of cobalt chloride and 1,6-hexanediol at low pH. The optimal condition is shown in Table 2 ▸. The crystals took approximately two weeks to grow to maximum size. Crystallization information is summarized in Table 2 ▸.

### 2.3. Data collection and processing   

Crystals were briefly soaked in the reservoir solution supplemented with 25% glycerol prior to data collection. Data were collected on beamline 24-ID-C at the Advanced Photon Source (APS), Argonne, Illinois, USA. Data were integrated using *XDS* (Kabsch, 2010 ▸) and were merged and scaled using *AIMLESS* (Evans, 2006 ▸). Data-collection and processing statistics are summarized in Table 3 ▸.

### 2.4. Structure solution and refinement   

An initial model of the crystal structure was determined using *ARCIMBOLDO* (Rodríguez *et al.*, 2009 ▸). Four partial helices were built in an automated fashion using the software, and the model was of sufficient quality for manual building into the electron-density map. In an iterative fashion, *ARCIMBOLDO_LITE* uses *Phaser* (molecular replacement) to search for short α-helices, followed by *SHELXE* to connect them into longer polypeptides. Following the last round of model building (rigid-body refinement) in *ARCIMBOLDO_LITE*, the final model consisted of 197 residues with a *Phaser* translation-function *Z*-score of 17.6 and a log-likelihood (LLG) score of 634 (Supplementary Fig. S1). The automated model building was remarkably successful: only five extra residues were built during further stages of manual refinement to give a total of 202 residues distributed over four α-helices. The additional residues and solutes (waters and hexanediol) were built by multiple rounds of model building and refinement through inspection of 2*F*_o_ − *F*_c_ maps using *Coot* (Emsley *et al.*, 2010 ▸) and *Phenix* (Liebschner *et al.*, 2019 ▸). *MolProbity* (Chen *et al.*, 2010 ▸) was used for Ramachandran analysis. Refinement statistics are summarized in Table 4 ▸.

## 3. Results and discussion   

**Figure 1:**
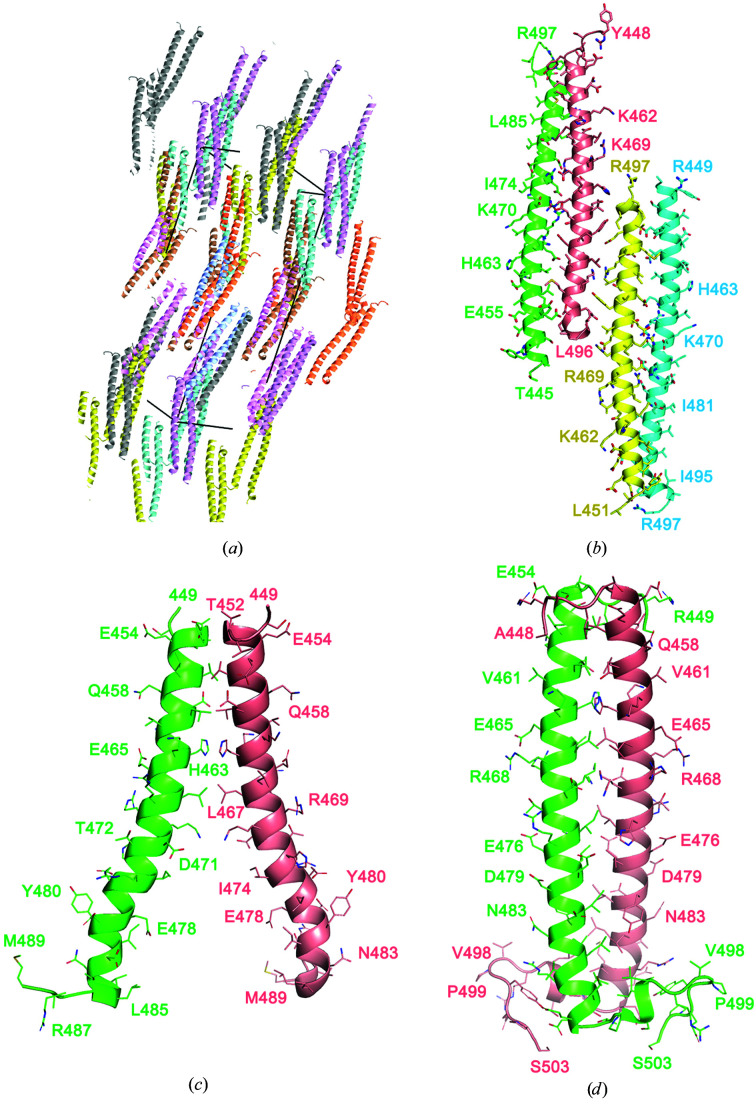
Crystal structure of uncomplexed FIP2. (*a*) Crystal packing in a section of the lattice. The asymmetric unit comprising four monomers is shown in identical colors. The monomers begin between residues 445 and 451, while the C-terminus is either residue 496 or 497. (*b*) The asymmetric unit consists of two pairs of antiparallel α-helices. (*c*) The NMR structure of uncomplexed FIP2 (residues 449–489) is a parallel dimer that is frayed as the helices extend towards the C-terminus. (*d*) The crystal structure of FIP2 (residues 448–503) from its complex with Rab11 (not shown). The closely packed parallel dimer has a hook at the C-terminus that is stabilized by hydrophobic packing.

Here, we describe an effector domain from a Rab11 family-interacting protein (FIP) in the uncomplexed state for the first time. Despite the known structures of FIP2 in complex with Rab11 and Rab25 (Jagoe, Lindsay *et al.*, 2006 ▸; Lall *et al.*, 2013 ▸), molecular replacement (MR) failed to provide a solution. Models of FIP2 from PDB entries 2gzd, 2gzh, 4c4p and 3tso were extracted and used as search models. All of these structures have a resolution of better than 2.5 Å. In order to account for possible flexibility at the termini, the core helical regions of FIP2 were searched as monomers and dimers, but did not provide clear solutions that produced interpretable electron-density maps. It is unclear why MR failed to distinguish correct solutions, at least for the monomeric α-helix. However, the crystal lattice consists exclusively of α-helices aligned with their long axes in similar orientations (Fig. 1 ▸ *a*). The nature of these crystals may pose a challenge for long α-helices as MR search models. Since crystal formation required the presence of a small amount of Co^2+^, data were also collected at the Co^2+^ absorption edge. Although there was a weak anomalous signal, it was insufficient for phasing of the structure. Wide-search molecular replacement from the full Protein Data Bank was performed using *Phaser* (McCoy *et al.*, 2007 ▸), as implemented in the Structural Biology Grid portal (https://sbgrid.org/; Stokes-Rees & Sliz, 2010 ▸). Candidate α-helical proteins were identified, but none were suitable as initial models for further refinement.

Phasing was successfully performed using *ARCIMBOLDO_LITE* as implemented within the *CCP*4 suite (Rodríguez *et al.*, 2009 ▸; Winn *et al.*, 2011 ▸). The crystal lattice of FIP2 can be described as an α-helical tetramer in which the long axes of the helices are coincident with the *c* axis of the crystal (Fig. 1 ▸ *a*). The asymmetric unit consists of two pairs of antiparallel α-helices that are assembled through polar interactions (Fig. 1 ▸ *b*). The polypeptides are of varying lengths depending on the extent of disorder at the N/C-termini. Although the segment 439–512 was subjected to crystallization, a minimum of the first seven residues and the last 15 residues are dis­ordered. The crystal structure of FIP2 is in contrast to the previously determined NMR solution structure of FIP2 (Fig. 1 ▸ *c*; Wei *et al.*, 2009 ▸) and the crystal structure of FIP2 in complex with Rab11 (Fig. 1 ▸ *d*; Jagoe, Jackson *et al.*, 2006 ▸). These structures are parallel α-helical dimers that are tightly associated by hydrophobic interactions.

**Figure 2:**
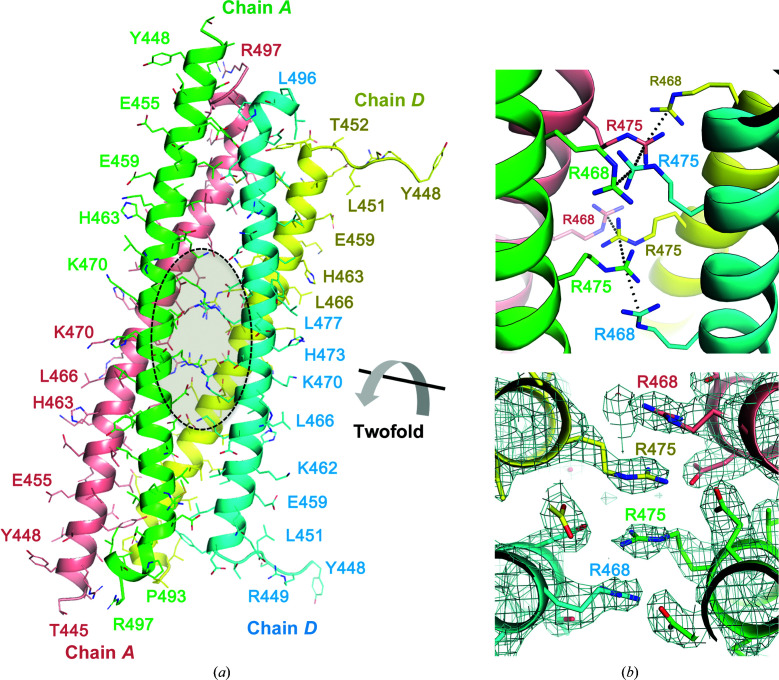
Arginine-stacking interactions stabilize a tetrameric FIP2 assembly. (*a*) The FIP2 tetramer in the lattice that is related by a twofold crystallographic axis. The axis runs through the middle of a stack of arginine residues that enable oligomerization into a four-helix bundle. (*b*) A view of the eight arginine residues from the four α-helices. The view is a close-up of the transparent ellipse in (*a*). (*c*) A section of the electron density (2*F*_o_ − *F*_c_, 1.5σ) within the region comprising the arginine-stacking interactions.

The packing interactions within the α-helical tetramer are fascinating and are worth closer inspection (Fig. 2 ▸). Dimers from the asymmetric unit form a stack of arginine residues with their symmetry-related dimers in the lattice (Fig. 2 ▸ *a*). Each helix contributes two arginines that form two layers of a four-arginine stack in the middle of the α-helical bundle (Fig. 2 ▸ *b*). The distances between the C^ζ^ atoms in the stacked guanidino side chains are 3.5–3.8 Å. Aspartate residues form salt bridges with these arginines and presumably contribute to orienting the guanidino groups (not shown). The electron-density map in this region reveals well ordered side chains (Fig. 2 ▸ *c*). Arg–Arg interactions have been recognized for their significant contributions to protein assemblies (Neves *et al.*, 2012 ▸; Vernon *et al.*, 2018 ▸). The distance between the stacks (<4 Å) is similar to the observed van der Waals distances from a survey of structures (Vernon *et al.*, 2018 ▸). However, the detailed energetics and stabilization of stacked arginines in protein assemblies are poorly characterized and require further study.

**Figure 3:**
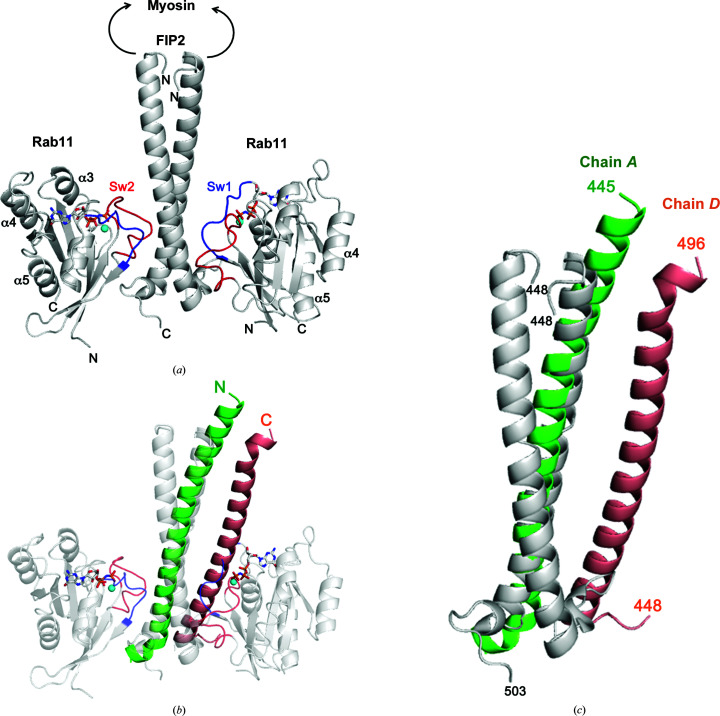
Comparisons of FIP2 in Rab11-bound and free states. (*a*) Complex of Rab11 (residues 7–173) with residues 448–503 of FIP2 (PDB entry 4c4p). The effector domain is at the extreme C-terminus of the 512-residue protein. Residues 129–290 of FIP2 (arrows), which lie upstream of the Rab11 effector domain, comprise the myosin Vb-binding region. Following α5 of Rab11, a hypervariable region of 43 residues is prenylated at two cysteine residues near the C-­terminus of the 216-residue protein. This flexible region was dispensed with to enable crystallization of the complex (Lall *et al.*, 2013 ▸). (*b*) Superposition of chain *A* of FIP2 with one of the α-helices from the Rab11–FIP2 complex. The parallel coiled coil is generated by a twofold symmetry operation from a 1:1 Rab11–FIP2 complex in the asymmetric unit. Therefore, the two α-helices in the complex are identical. The Rab11–FIP2 ribbons are displayed with transparency. (*c*) Superposition of the α-helices of FIP2. Identical segments of FIP2 were aligned by a secondary-structure matching algorithm using *Coot*. The 39 core residues in the α-helices aligned with a root-mean-square (r.m.s.) deviation of 1.8 A for their C^α^ atoms.

A physiological model for complex formation involves Rab11 recruitment of pre-formed parallel dimers of cytosolic FIP2 to endosomes (Jagoe, Lindsay *et al.*, 2006 ▸; Eathiraj *et al.*, 2006 ▸). This model is premised on the finding that switch 1 and switch 2 of a single Rab11 molecule interact with both α-helices of FIP2 in a symmetric fashion to form a heterotetrameric complex (Fig. 3 ▸ *a*). Also, FIP2 spontaneously forms dimers in solution at physiological pH (Jagoe, Jackson *et al.*, 2006 ▸; Jagoe, Lindsay *et al.*, 2006 ▸). It is probable that parallel dimers dissociate to form the crystals observed here at pH 4.8. Therefore, the physiological relevance of the structure of uncomplexed FIP2 observed under these conditions is unknown. Nevertheless, it is intriguing to observe the conformational flexibility of the α-helices of FIP2 under various conditions. Although the central α-helical regions are similar in all structures, the N- and C-termini diverge significantly (Figs. 3 ▸ *b* and 3 ▸ *c*). Whether these variations reflect possible dynamic changes of FIP2 during membrane trafficking requires further investigation. Interestingly, crystals of the Rab11–FIP2 complex have been grown at pH 4.5 (Jagoe, Lindsay *et al.*, 2006 ▸), albeit under different precipitant conditions. It is conceivable that FIP2 can exist in multiple conformational states and that Rab11 binds selectively to the parallel dimer during the crystallization process.

In summary, the uncomplexed structure of FIP2 reveals head-to-tail oligomers of α-helices that are stabilized by polar interactions. The diffraction data and crystal structure may contribute to a useful archive for further improvement of techniques in macromolecular phasing.

## Supplementary Material

PDB reference: Rab11 family-interacting protein 2, 6s8x

Supplementary Figure S1. DOI: 10.1107/S2053230X20009164/ft5109sup1.pdf

## Figures and Tables

**Table 1:** Macromolecule-production information

Source organism	*Homo sapiens*
Forward primer	TACTTCCAATCCATGAGCAACCCCTTTGATGCCACTGCA
Reverse primer	TATCCACCTTTACTGTTAACTGTTAGAGAATTTGCCAGCTTTCCT
Cloning vector	MBP-FIP2 construct prepared as in Jagoe, Lindsay *et al.* (2006 ▸)
Expression vector	pNIC-Bsa4 (a variant of pET-28b containing a TEV protease cleavage site)
Expression host	*Escherichia coli* BL21(DE3)
Complete amino-acid sequence of the construct produced	GSHMSNPFDATAGYRSLTYEEVLQELVKHKELLRRKDTHIRELEDYIDNLLVRVMEETPSILRVPYEPSRKAGKFSNS

**Table 2:** Crystallization

Method	Vapor diffusion
Plate type	Linbro
Temperature (K)	298
Protein concentration (mg ml^−1^)	6
Buffer composition of protein solution	150 m*M* NaCl, 10 m*M* Tris–HCl, 1 m*M* DTT pH 7.5
Composition of reservoir solution	0.01 *M* cobalt chloride, 0.1 *M* sodium acetate pH 4.8, 1 *M* 1,6-hexanediol
Volume and ratio of drop	1 µl:1 µl
Volume of reservoir (µl)	500

**Table 3:** Data collection and processing Values in parentheses are for the outer shell.

Diffraction source	Beamline 24-ID-C, APS
Wavelength (Å)	0.9791
Temperature (K)	100
Detector	Dectris PILATUS 6M-F
Space group	*C*222_1_
*a*, *b*, *c* (Å)	62.54, 68.43, 172.09
α, β, γ (°)	90, 90, 90
Resolution range (Å)	46.16–2.29
Total No. of reflections	72768
No. of unique reflections	16885
Completeness (%)	99.13
Multiplicity	4.3
〈*I*/σ(*I*)〉	8.8
Overall *B* factor from Wilson plot (Å^2^)	35.97

**Table 4:** Structure refinement Values in parentheses are for the outer shell.

Resolution range (Å)	43.022–2.290
Completeness (%)	99.1
No. of reflections, working set	16885
No. of reflections, test set	824
Final *R*_cryst_	0.246
Final *R*_free_	0.274
No. of non-H atoms	
Total	1791
Protein	1721
Ligand	8
Water	62
R.m.s. deviations	
Bond lengths (Å)	0.007
Angles (°)	1.05
Average *B* factors (Å^2^)	
Overall	45.36
Protein	45.09
Ligand	47.23
Water	52.67
Ramachandran plot	
Favored regions (%)	100
