# Supplementary material for: Crystal structure of the Rab-binding domain of Rab11 family-interacting protein 2
Source: Acta Crystallogr F Struct Biol Commun. 2020 Jul 28;76(Pt 8):357–63. doi: 10.1107/S2053230X20009164 (PMC7397465; doi:10.1107/S2053230X20009164)
Supplement: Supplementary file 1 [file f-76-00357-sup1.pdf]

STRUCTURAL BIOLOGY
COMMUNICATIONS

Volume 76 (2020)

Supporting information for article:

Crystal structure of the Rab-binding domain of Rab11 family-interacting protein 2

Aoife Mairead Kearney and Amir Rafiq Khan

2. STEP: Locating Sequentially the Fragments

Fragment 1

Cluster	Rotation Function					Translation Function					Packing					Rigid Body Refinement					Initial CC	Best Trace CC/aa		
	#Rots.	Top LLG	Mean LLG	Top Zscore	Mean Zscore	#Trans.	Top LLG	Mean LLG	Top Zscore	Mean Zscore	#Sol.	Top LLG	Mean LLG	Top Zscore	Mean Zscore	#Sol.	Top LLG	Mean LLG	Top TFZ==	Mean TFZ==	After Refinement CC	Cycle	CC	#Res. traced
0	190	87.31	72.13	4.22	3.58	91	102.79	99.78	5.94	5.02	21	102.79	99.61	5.38	4.84	42	107.50	97.77	6.10	4.65	16.84			
1	181	86.93	72.67	4.21	3.60	76	103.90	99.60	7.03	5.32	23	103.90	99.75	6.30	5.28	43	111.40	97.53	6.30	4.58	16.56			

Fragment 2

Cluster	Rotation Function					Translation Function					Packing					Rigid Body Refinement					Initial CC	Best Trace CC/aa		
	#Rots.	Top LLG	Mean LLG	Top Zscore	Mean Zscore	#Trans.	Top LLG	Mean LLG	Top Zscore	Mean Zscore	#Sol.	Top LLG	Mean LLG	Top Zscore	Mean Zscore	#Sol.	Top LLG	Mean LLG	Top TFZ==	Mean TFZ==	After Refinement CC	Cycle	CC	#Res. traced
(0, 0)	300	177.54	172.45	3.99	3.81	300	205.17	188.94	8.22	6.55	8	205.17	201.00	8.22	7.75	29	215.20	183.39	10.10	7.12	19.33			
(0, 3)	300	179.15	172.91	4.07	3.84	300	226.29	186.97	10.00	6.55	93	226.29	206.53	10.00	8.22	150	261.20	209.67	13.10	9.36	20.43			
(1, 1)	300	182.78	175.15	4.12	3.80	300	221.93	205.39	9.53	7.83	68	221.93	209.28	9.42	8.18	150	252.80	203.68	12.70	9.01	20.45			
(1, 2)	300	181.44	174.81	4.03	3.77	300	241.30	201.36	10.50	7.50	102	241.30	209.68	10.50	8.24	150	257.40	210.92	12.60	9.29	20.63			

Fragment 3

Cluster	Rotation Function					Translation Function					Packing					Rigid Body Refinement					Initial CC	Best Trace CC/aa		
	#Rots.	Top LLG	Mean LLG	Top Zscore	Mean Zscore	#Trans.	Top LLG	Mean LLG	Top Zscore	Mean Zscore	#Sol.	Top LLG	Mean LLG	Top Zscore	Mean Zscore	#Sol.	Top LLG	Mean LLG	Top TFZ==	Mean TFZ==	After Refinement CC	Cycle	CC	#Res. traced
(0, 0, 3)	300	323.05	314.73	4.00	3.70	226	383.05	357.87	11.13	9.57	226	383.05	357.87	11.13	9.57	150	404.10	359.88	13.80	10.51	24.51			
(0, 3, 3)	300	323.19	314.92	4.03	3.70	211	405.87	361.90	13.05	9.78	211	405.87	361.90	13.05	9.78	150	423.20	377.79	14.70	11.81	24.82			
(1, 1, 1)	300	315.13	304.89	3.97	3.59	278	415.65	363.40	14.86	10.64	274	415.65	363.60	14.86	10.64	150	429.70	370.63	15.90	12.63	25.39			
(1, 1, 2)	300	319.53	311.61	3.99	3.68	157	412.33	362.76	13.69	10.06	157	412.33	362.76	13.69	10.06	150	426.20	374.46	15.50	11.99	24.75			
(1, 1, 5)	300	314.99	297.74	4.02	3.65	188	396.46	359.76	13.24	10.81	188	396.46	359.76	13.24	10.81	150	426.40	373.34	15.70	13.33	24.67			
(1, 2, 2)	300	319.66	311.45	4.00	3.67	227	386.09	357.14	11.53	9.77	227	386.09	357.14	11.53	9.77	150	408.80	364.36	14.10	11.08	24.56			

Fragment 4

Cluster	Rotation Function					Translation Function					Packing					Rigid Body Refinement					Initial CC	Best Trace CC/aa		
	#Rots.	Top LLG	Mean LLG	Top Zscore	Mean Zscore	#Trans.	Top LLG	Mean LLG	Top Zscore	Mean Zscore	#Sol.	Top LLG	Mean LLG	Top Zscore	Mean Zscore	#Sol.	Top LLG	Mean LLG	Top TFZ==	Mean TFZ==	After Refinement CC	Cycle	CC	#Res. traced
(0, 0, 3, 3)	300	478.56	468.43	3.97	3.68	300	604.42	546.97	15.39	12.27	300	604.42	546.97	15.39	12.27	150	626.40	532.39	18.00	14.21	29.57			
(0, 3, 3, 3)	300	479.79	468.53	4.06	3.69	201	598.81	533.25	15.22	11.58	201	598.81	533.25	15.22	11.58	150	622.00	527.86	17.90	13.91	29.59			
(1, 1, 1, 1)	300	484.21	476.58	3.97	3.59	300	615.03	552.06	15.82	12.21	299	615.03	552.18	15.82	12.21	150	634.00	544.51	17.60	14.40	30.11	5	49.99	197
(1, 1, 1, 2)	300	479.11	468.95	4.00	3.41	41	584.57	514.05	14.73	10.47	34	584.57	516.46	14.73	10.61	150	627.20	508.39	18.80	13.36	29.72			
(1, 1, 1, 5)	300	478.53	469.19	4.01	3.49	16	509.01	502.45	10.36	9.62	12	509.01	502.81	9.79	9.46	150	627.00	437.93	17.70	12.01	29.76			
(1, 1, 2, 2)	300	477.08	469.03	3.94	3.41	300	605.30	554.21	15.67	12.70	300	605.30	554.21	15.67	12.70	150	628.00	542.29	18.40	16.76	29.86			

Show All Hide Not Relevant

The current best solution is: ensembleIDxx2FR3_16-3.pdb with FINALCC: 49.99 and n. residues traced 197
file is: /labdata/rglab/akhan/Documents/FIP2_structure/1_arcimboldo_lite/8_EXP_LIBRARY/5/0/ensembleIDxx2FR3_16-3.pdb

- FRF: Pos. in Rank: **37** LLG: **480.26** ZSCORE: **3.71** Top LLG in Cluster **(1, 1, 1, 1)**: **484.21** Top ZSCORE in Cluster **(1, 1, 1, 1)**: **4.06**
- REFINEMENT ROTATION AND MODEL

- FTF: Pos. in Rank: **8** LLG: **605.77** ZSCORE: **14.88** Top LLG in Cluster **(1, 1, 1, 1)**: **615.03** Top ZSCORE in Cluster **(1, 1, 1, 1)**: **15.82**
- PACK: Pos. in Rank: **8** LLG: **605.77** ZSCORE: **14.88** Top LLG in Cluster **(1, 1, 1, 1)**: **615.03** Top ZSCORE in Cluster **(1, 1, 1, 1)**: **15.82**
- RNP: Pos. in Rank: **5** LLG: **631.40** ZSCORE: **14.88** TFZ==: **17.20** Top LLG in Cluster **(1, 1, 1, 1)**: **634.00** Top ZSCORE in Cluster **(1, 1, 1, 1)**: **15.82**
- INITIAL CC
    - After Refinement: Pos. in Rank: **4** INITCC: **30.04** Top INITCC in Cluster **(1, 1, 1, 1)**: **30.11**
- EXPANSION

Cycle 5:
Final CC: 49.99% N. Residues Traced: **197.00**

Figure S1 Results of phasing from ARCIMBOLDO_LITE.
